# Region-Specific Alterations of Perineuronal Net Expression in Postmortem Autism Brain Tissue

**DOI:** 10.3389/fnmol.2022.838918

**Published:** 2022-04-13

**Authors:** Cheryl Brandenburg, Gene J. Blatt

**Affiliations:** ^1^Hussman Institute for Autism, Baltimore, MD, United States; ^2^Department of Pharmacology, University of Maryland School of Medicine, Baltimore, MD, United States

**Keywords:** perineuronal net (PNN), dentate nucleus of the cerebellum, globus pallidus (GP), autism spectrum disoder (ASD), parvalbumin (PV)

## Abstract

Genetic variance in autism spectrum disorder (ASD) is often associated with mechanisms that broadly fall into the category of neuroplasticity. Parvalbumin positive neurons and their surrounding perineuronal nets (PNNs) are important factors in critical period plasticity and have both been implicated in ASD. PNNs are found in high density within output structures of the cerebellum and basal ganglia, two regions that are densely connected to many other brain areas and have the potential to participate in the diverse array of symptoms present in an ASD diagnosis. The dentate nucleus (DN) and globus pallidus (GP) were therefore assessed for differences in PNN expression in human postmortem ASD brain tissue. While Purkinje cell loss is a consistent neuropathological finding in ASD, in this cohort, the Purkinje cell targets within the DN did not show differences in number of cells with or without a PNN. However, the density of parvalbumin positive neurons with a PNN were significantly reduced in the GP internus and externus of ASD cases, which was not dependent on seizure status. It is unclear whether these alterations manifest during development or are a consequence of activity-dependent mechanisms that lead to altered network dynamics later in life.

## Introduction

The most recent estimate of autism spectrum disorder (ASD) prevalence rose to 1 in 44 children (Walensky et al., 2021), an 18% increase from the previous rate of 1 in 54 announced by the Centers for Disease Control and Prevention (Redfield et al., 2020). With this steady increase in ASD prevalence each year, it is becoming increasingly important to identify the underlying neurodevelopmental mechanisms that contribute to ASD symptoms. Although the brain regions involved and the molecular underpinnings implicated in ASD are diverse, a diagnosis centers on social communication and sensorimotor challenges within the domain of restricted and repetitive behaviors (American Psychiatric Association, 2013). As many complex behaviors, such as language, require the integration of information from visual, auditory, tactile, and motor systems, it has been proposed that people with ASD struggle to unify multisensory information into a single percept (Baum et al., 2015; Chan et al., 2016; Stevenson et al., 2016; Robertson and Baron-Cohen, 2017; Beker et al., 2018; Zhou et al., 2018). As such, brain plasticity mechanisms are implicated in ASD and have driven research into vulnerable aspects of critical period plasticity (Wang et al., 2014; Yousif Ismail et al., 2016; Hansel, 2018; Reh et al., 2020) as well as its relationship to altered network dynamics and excitatory/inhibitory imbalance (Blatt et al., 2001; Hussman, 2001; Rubenstein and Merzenich, 2003; Gogolla et al., 2009; Levin et al., 2017; Gabard-Durnam et al., 2019).

The disruptions in critical period plasticity may lead to altered network dynamics later in life and have been connected to the function of parvalbumin (PV) positive interneurons (Reh et al., 2020). As recently reviewed in Ruden et al. (2021), PV interneurons are particularly vulnerable to a diverse range of stressors and have been linked to ASD. While the exact timing may differ between brain regions and cell-types, the closure of the critical period of plasticity coincides with the formation of perineuronal nets (PNNs) that surround the soma and proximal dendrites of PV interneurons (Cabungcal et al., 2013; Fawcett et al., 2019; Reichelt et al., 2019).

Perineuronal nets (for recent general review refer to: Shen, 2018; Bosiacki et al., 2019; Burket et al., 2021; Carulli and Verhaagen, 2021; Jakovljević et al., 2021) are a specialized and condensed form of extracellular matrix (ECM), which are consistently found to have genetic susceptibility in ASD from genome wide association studies (GWAS) (Wang et al., 2009; Weiss and Arking, 2009; Anney et al., 2010; Hussman et al., 2011). Among these genes, Reelin, a disintegrin and metalloproteinase with thrombospondin motifs (ADAMTS) and semaphorins are some of the strongest evidence linking ASD to disruption of the ECM (for review see: Pantazopoulos and Berretta, 2016; Sorg et al., 2016). Additionally, studies of PNNs have been prominent in animal models of fragile X syndrome, where reduced PNN expression has been demonstrated in Fmr1 knockout mouse auditory cortex and amygdala with relevance to altered fear-associated memory (Reinhard et al., 2019). In another study, pharmacological inhibition or genetic reduction of matrix component metalloproteinase-9 results in increased PNN production surrounding PV neurons, which normalizes auditory deficits in Fmr1 knockout mice (Wen et al., 2018; Pirbhoy et al., 2020). Xia et al. (2021) utilized a valproic acid (VPA) mouse model of ASD and found differences in intensities of PV and PNN subpopulations that possibly contribute to the progression of ASD. While these few studies show potential in exploring PNN function in relationship to ASD, the literature is lacking on specific mechanisms that may link PNNs to relevant behaviors and whether the GWAS mutations reported lead to PNN dysfunction. It is unclear whether a connection of PNNs to ASD definitively exists and, if so, whether or not the PNN changes may be a primary cause or a secondary effect due to other processes in brain development.

In contrast to animal model studies, there is a lack of literature on PNN distribution in postmortem samples from patients with idiopathic autism. Thus, the current investigation centered around regions of the brain with high PNN density and relevance to ASD-related behaviors. Postmortem brain samples were taken from both the dentate nucleus (DN) of the cerebellum that has high PNN expression (Blosa et al., 2016; Carulli et al., 2020; Hirono et al., 2021) as well as the globus pallidus (GP) of the basal ganglia (Adams et al., 2001; Cabungcal et al., 2019). The DN and GP are sources of outgoing projections to the thalamic, motor, premotor, and sensory cortices that can affect the functionality of excitatory cortical neurons and play critical roles in many ASD-related cognitive, sensory and motor behaviors. Therefore, PNN expression in these two critical regions was quantified in ASD compared to neurotypical samples. While the DN did not show differences in neuronal number or PNN expression, the GP had significantly reduced PNN expression in ASD cases that was not dependent on seizure status. Future studies may aim to clarify the role PNN and PV neurons may play in plasticity and, ultimately, an ASD diagnosis.

## Methods

### Postmortem Tissue

Human postmortem brain tissue was obtained from the University of Maryland Brain and Tissue Bank, a brain and tissue repository of the NIH Neurobiobank. ASD cases were confirmed through Autism Diagnostic Interview-Revised (ADI-R) scores and/or received a clinical diagnosis of ASD from a licensed psychiatrist, case demographics are provided in Table 1. The University of Maryland Brain and Tissue Bank (NIH Neurobiobank) is overseen by Institutional Review Board protocol number HM-HP-00042077 and de-identifies all cases before distribution to researchers.

**TABLE 1:** Postmortem tissue case demographics.

Cases	GP	DN	Diagnosis	Age	PMI	Gender	Ethnicity	Cause of death
914	x	x	Control	20	18	M	Caucasian	Vehicle accident, multiple injuries
1158	x	x	Control	16	15	M	Caucasian	Cardiomegaly
4337	x	x	Control	8	16	M	African American	Blunt force neck injury
4599	x		Control	23	18	M	African American	Cardiac arrhythmia/anomalous coronary artery
4787	x	x	Control	12	15	M	African American	Asthma
5030	x		Control	24	14	M	African American	Reactive airway disease
5113	x	x	Control	36	20	M	African American	Pulmonary embolism
5334	x	x	Control	12	15	M	Hispanic	Hanging/suicide
5376	x	x	Control	13	19	M	Caucasian	Hanging
5387	x	x	Control	12	13	M	Caucasian	Drowning
5566	x	x	Control	15	23	F	African American	Hypertrophic cardiomyopathy
5646	x	x	Control	20	23	F	Caucasian	Reactive airway disease
5669	x	x	Control	24	29	F	African American	Hypertensive cardiovascular disease
5705	x	x	Control	31	26	M	Caucasian	Cardiac arrhythmia
5759	x	x	Control	34	28	M	Caucasian	Atherosclerotic cardiovascular disease
5813	x	x	Control	20	24	M	African American	Atherosclerotic cardiovascular disease
5889	x	x	Control	27	12	M	Caucasian	Acute pneumonia complicated by sepsis
5893	x	x	Control	19	11	M	Caucasian	Dilated cardiomegaly
5922	x	x	Control	46	10	M	Caucasian	Atherosclerotic cardiovascular disease
5926	x	x	Control	21	27	M	African American	Cardiac arrhythmia with probable sickle cell disease
5958	x	x	Control	22	24	M	African American	Dilated cardiomegaly
3916	x	x	Autism	32	22	M	Caucasian	Congestive heart failure
4334	x	x	Autism	11	27	M	Hispanic	Acute hemorrhagic tracheobronchitis
4899	x		Autism	14	9	M	Caucasian	Drowning
5027	x	x	Autism	37	26	M	African American	Obstruction of bowel due to adhesion
5115*	x	x	Autism	46	29	M	Caucasian	Complications of pseudomyxoma peritonei
5144	x	x	Autism	7	3	M	Caucasian	Cancer
5176	x	x	Autism	22	18	M	African American	Subdural hemorrhage
5278*	x		Autism	15	13	F	Caucasian	Drowning associated with seizure disorder
5403	x	x	Autism	16	35	M	Caucasian	Cardiac arrhythmia
5419*	x	x	Autism	19	22	F	Caucasian	Natural/epilepsy
5565*		x	Autism	12	22	M	African American	Seizure disorder, complications
5574	x		Autism	23	14	M	African American	Pneumonia
5631	x		Autism	18	96	M	Caucasian	Acute hepatic failure
5771	x	x	Autism	27	5	F	Caucasian	Undetermined
5841	x	x	Autism	12	15	M	Caucasian	Hanging
5864*	x	x	Autism	20	42	M	Caucasian	Seizure disorder
5878	x	x	Autism	27	42	M	Caucasian	Peritonitis
5939*	x	x	Autism	21	22	M	Caucasian	Cardiovascular related
5940*	x	x	Autism	29	20	M	Caucasian	Epilepsy complicated by drowning
5945*	x	x	Autism	20	24	M	Caucasian	Chronic pulmonary aspergillosis
5978	x	x	Autism	11	21	M	Caucasian	Smoke inhalation
6033*		x	Autism	14	25	F	Caucasian	Seizure disorder

*At least one documented seizure.

Nineteen control and 18 ASD formalin fixed age-, gender-, and PMI-matched human DN blocks were dissected in a consistent anatomical location across cases. Similarly, blocks of GP were dissected so that both the GPe and the GPi were contained within the same block for a total of 21 control and 20 ASD cases. For the DN, there are potential differences in the exact anatomical levels that were dissected between cases. For the GP, anatomical levels were highly consistent because all blocks contained both GPi and GPe within one section, therefore, they could only be dissected for the stretch of tissue where both areas are prominent. Blocks of DN and GP were chosen from the same cases where possible (Table 1). Blocks were rinsed, cryoprotected, flash frozen and stored at −80^°^C until they were cut at 40 μm, in series, onto glass slides with a cryostat (Leica CM1950) and again frozen at −80^°^C.

### Immunohistochemistry

Immunohistochemistry (IHC) was performed similarly to Hoffman et al. (2016). Five frozen DN sections (every sixth interval- spanning a total of 1,200 μm) on slides from each case were thawed, dipped in KPBS and dried on a slide drying rack before antigen retrieval in tris buffer (pH 9.0) in a preheated scientific microwave (Ted Pella) at 35°C, 150 W for 10 min. Sections were then placed in 1% hydrogen peroxide in KPBS for 20 min at room temperature. Three washes in KPBS at 35°C and 150 W for 1 min were performed (all subsequent wash steps are performed in this manner). Non-specific blocking for 30 min in 8% horse serum in KPBS was completed before incubation in primary antibody (anti-HPLN1 1:150, R&D Systems 2608-HP) for 48 h at 4°C. Sections were washed and placed in biotinylated secondary antibody (anti-goat 1:700, Vector Laboratories BA-9500) for 1 h then washed again. After incubation in avidin-biotin complex (A/B) (Vector Laboratories PK-6100) for 1 h, sections were rinsed first in KPBS then in 0.175 M sodium acetate before a 20 min exposure to nickel (Sigma SIG-N4882) 3 3′-diaminobenzidine tetrahydrochloride hydrate (DAB) (Sigma SIG-32750) in sodium acetate. Sections were washed in sodium acetate and then KPBS before being dipped in distilled water and incubated in 1% neutral red (Sigma) for 30 min and subsequently run through a series of alcohol dehydrations. Sections were placed in xylene (SIG-534056) for 8 min then mounted with DPX (Sigma SIG-06522) and coverslipped.

In the GP, all steps were the same, except after the first nickel DAB reaction with HPLN1, slides were washed then the primary step was repeated for 48 h with anti-PV (1:300, Sigma P3088) and processed in the same manner with biotinylated anti-mouse secondary (1:600, Vector Laboratories BA-2000), but nickel was not included in the second DAB reaction to produce a brown reaction product instead of black. Neutral red was omitted on these sections. All sections, from both control and ASD for each region, were run in parallel using the same initial solutions to limit variability in processing.

### Imaging and Quantification

**FIGURE 1:**
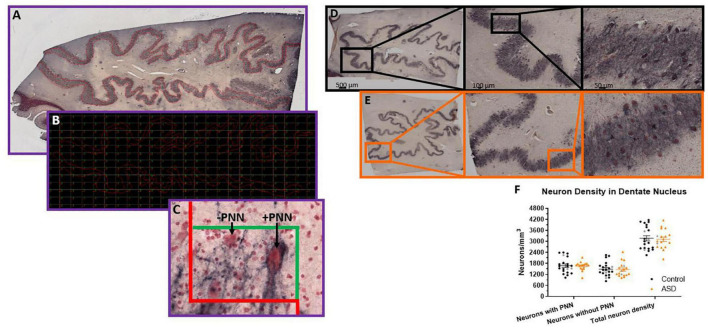
Stereological setup for quantifying perineuronal net density in human postmortem tissue. Contours were manually drawn over the region of interest, in this case **(A)**, the dentate nucleus of the cerebellum. Contours were placed at a consistent distance from the edge of the dentate to mainly capture the high perineuronal net density locations in the center of the ribbon-like region. Therefore, the boxes actually counted within the grid placed over the contour in the software **(B)** would only be areas with a high density of neurons. Unstained regions are not represented. In this manner, unbiased counts can be achieved following established stereological methods, where neurons falling on the red line of the counting box **(C)** are not counted and any neurons falling within the box or on the green line are counted. Arrows point to examples of a dentate neuron surrounded by a perineuronal net and an example of a neuron stained with neutral red, but not surrounded by a perineuronal net. The neuron in the lower left would not be counted because it falls on the dissector red line, but otherwise would be PNN positive since it has an outline of black around the soma (albeit lighter than the other cell) and dark proximal axon area. **(D)** Immunohistochemistry of HPLN1 to visualize neurons surrounded by a perineuronal net while all neurons are stained with neutral red in a control **(D)** and ASD **(E)** case of the dentate nucleus within the cerebellum. **(F)** The density of neurons surrounded by a perineuronal net (PNN), without a PNN and therefore the total density of all neurons within the dentate were not significantly different between control and ASD cases, measured by a Student’s *t*-test. Females in each group are identified as gray colored symbols.

A Zeiss Microbrightfield Stereoinvestigator system was used to quantify neuronal densities within the manually drawn contours. In the DN, contours were drawn just inside the border where neuron density was highest and excluded regions without strong staining, which created a ribbon-like outline (Figure 1). Only a small percentage (<5%) of each DN section had light staining (typically near the very edge of the tissue) so should not affect overall density counts. The density of neurons surrounded by a PNN, neurons without a PNN and total neuron numbers were estimated with the optical fractionator method then divided by the total estimated area using the Cavalieri method. The GP was quantified in a similar manner except that one circular contour contained the entire region of interest and neurons were only counted if they had both a PNN and a PV stained soma. This region did not show variability in staining and was typically surrounded by white matter so that edge effects were less of an issue. Therefore, the entire visible GP region was encircled and the counting grid was randomly placed over the entire area.

The Stereoinvestigator software has standardized programs to input counting grid sizes for the desired counting box coverage and automatically calculate neuron density following stereological principles (Gundersen, 1986; West et al., 1991). The counting grid size was adjusted until the number of boxes counted averaged 100–200 in the DN and 350–450 in each region of the GP of five 40 μm sections in each case series (every 6th interval), resulting in a dissector volume of 0.00095 mm^3^. The dissector height was set at 30 μm, leaving guard zones of roughly 5 μm of the 40 μm sections, depending on tissue shrinkage differences. While the sections always contained enough tissue for the dissector height and tissue above and below while measuring at individual sites, it is possible that tissue shrinkage may lead to partial cells counted in some instances. Investigators were blinded to the diagnosis until after all density counts were completed.

### Statistical Analyses

Neuron density, calculated by the Cavalieri method within the Stereoinvestigator software, was plotted (control vs. ASD) for each region and a Student’s *t*-test was performed using GraphPad Prism 7. Regression analyses were carried out in Microsoft Excel (2013) and fitted with trendlines to obtain R^2^ values. GraphPad Prism 7 was utilized to compare the slope and intercept (elevation) of the trendlines, which is equivalent to an analysis of covariance (ANCOVA). All tissue available was quantified and tests for outliers were not performed.

## Results

Given the reported PC reductions in ASD (Bauman and Kemper, 1985; Bauman et al., 1995; Bailey et al., 1998; Whitney et al., 2008; Schumann and Nordahl, 2011; Skefos et al., 2014; Hampson and Blatt, 2015), we aimed to determine whether there are further deficits in neuronal numbers within the cerebellar circuitry, as DN neurons are likely to be impacted by PC deficits. PNNs around the DN neurons were also quantified as a readout for disrupted activity from PCs using hyaluronan and proteoglycan link protein 1 (HPLN1). HPLN1 is a critical component of PNN formation and has been shown to affect critical period plasticity after knockout (Carulli et al., 2010). Following stereological principles for estimating number within a volume, five sections for each case had a contour manually drawn around each region of interest (Figures 1A–C). The counting grid was adjusted to produce an average of 150 counting boxes per case and the total number of neurons with or without a PNN were totaled then divided by the number of counting sites times the dissector volume (0.00095 mm^3^).

The density of neurons (Figures 1D–F) with PNNs, based on HPLN1 expression, was not different (control mean = 1,682.27 ± 417.51 and autism mean = 1,658.57 ± 228.93 neurons/mm^3^, *p* = 0.83). The density of neurons without a PNN also showed no differences (control mean = 1,487.74 ± 386.44 and autism mean = 1,462.14 ± 384.31 neurons/mm^3^, *p* = 0.84) and therefore total neuron numbers were similar (control mean = 3,170.01 ± 632.64 and autism mean = 3,120.71 ± 520.12 neurons/mm^3^, *p* = 0.79).

**FIGURE 2:**
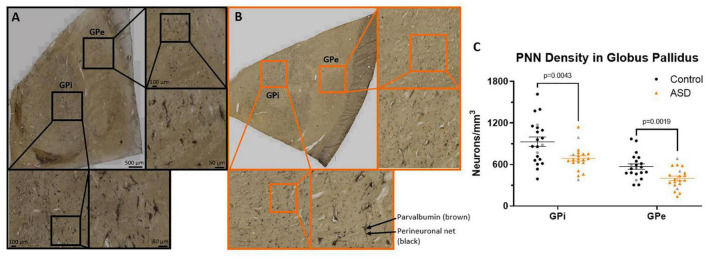
Immunohistochemistry of HPLN1 (black) and parvalbumin (brown) to visualize inhibitory projection neurons surrounded by a perineuronal in a control **(A)** and ASD **(B)** case of the globus pallidus (GP). The entire visible region of the GPi and GPe were each included in the contour manually drawn for inclusion within the counting grid. **(C)** The density of parvalbumin positive neurons surrounded by a perineuronal net were significantly decreased in ASD cases compared to controls in both the GPi and GPe using a Student’s *t*-test. Females in each group are identified as gray colored symbols.

The shapes of the GPi and GPe (Figure 2A) allowed for circling the entire region of interest with one contour, with counting boxes filling the center. The same grid box size was utilized as for the DN, except the number of counting sites averaged 370 for the GPi and 442 for the GPe across the five sections in series of every 6th interval. In pilot studies, there were not a significant portion of neurons that were either PV positive or PNN positive only, therefore, only neurons with a clear PNN and PV filled in the center were counted. PNN stain could be seen throughout the tissue as black “lines” following neuronal processes, but only somas with PV staining were counted as separate neurons.

Neurons throughout the GPi and GPe that stained with both HPLN1 and PV were quantified and compared by diagnosis (Figure 2C), resulting in significant decreases in the ASD cases within the GPi (control mean 929.91 ± 312.81 and autism mean 687.96 ± 174.95, *p* = 0.0043) and the GPe (control mean 570.46 ± 178.85 and autism mean 399.54 ± 146.89, *p* = 0.0019).

**FIGURE 3:**
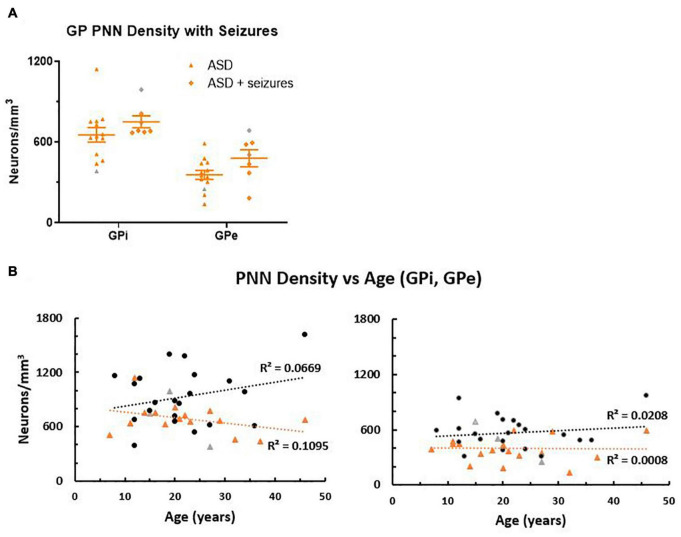
**(A)** Autism spectrum disorder cases were separated into groups based on medical reports of seizure occurrence. In the globus pallidus (GP), all seven seizure cases had a diagnosis of epilepsy. Although the group means were not statistically different, the seizure cases trended toward higher perineuronal net density than the ASD cases with no history of seizures. **(B)** Perineuronal net density was plotted against age in both the GPi and GPe, which showed a positive correlation of density with age in the control group, but a negative correlation with age in the ASD group in the GPi, which was less pronounced in the GPe. However, slopes of the lines were not significantly different (GPi *p* = 0.093; GPe *p* = 0.57), but intercept (elevation) was significantly decreased in ASD for both the GPi (*p* = 0.0048) and the GPe (*p* = 0.0022) using a GraphPad Prism 7 model equivalent to analysis of covariance (ANCOVA), reflecting the decreased PNN density as a group. Females are identified as gray colored symbols.

In animal studies, degradation of PNNs occurs after seizures (Mcrae et al., 2012; Rankin-Gee et al., 2015; Yutsudo and Kitagawa, 2015) so the reduction of PNNs in the GP of ASD cases could be a result of seizure activity instead of a common feature across individuals. Within this cohort, 7/20 ASD cases also had a diagnosis of epilepsy, but comparing group means (Figure 3A) showed that the cases with seizures were not driving the decreases in lower PNNs for the ASD group as a whole. Instead, there was a trend toward higher PNN density in the seizure cases compared to the ASD only cases that did not reach significance (GPi *p* = 0.24, GPe *p* = 0.07).

Perineuronal net density increases with age in human postmortem samples (Rogers et al., 2018). Therefore, PNN density was compared to age (Figure 3B), which showed a tendency to increase with age in controls, but was slightly decreased in ASD within the GPi. However, the difference between slopes measured by a Student’s *t*-test was not quite significant (*p* = 0.093). Cases had relatively the same slope across age in the GPe (*p* = 0.57). Intercepts of the line, representing PNN density between groups, were significantly decreased in ASD cases for both the GPi (*p* = 0.0048) and the GPe (*p* = 0.0022).

Since the numbers of PNNs in ASD tissue were lower and the tissue often appeared to have a generally lighter stain (Figure 2), fresh frozen tissue from the opposite hemisphere from the same cases were assessed with western blotting in an attempt to quantify decreases in PNN component levels. However, these data were unreliable, as individual components of nets that should increase or decrease together, such as brevican and HPLN1, were highly variable even when measured from the same case on the same blot. Therefore, we did not include the results from these analyses, as discussed in the section “Limitations”.

## Limitations

Although our number of cases included for comparisons is significantly higher than typically used in postmortem studies, the variability inherent to tissue processing and individual variation makes interpretation challenging. This is especially true for PNN analyses because, as other researchers have noted, variable fixation practices after brain collection, differences in collection strategies and the difficulties of extracting PNN components from surrounding tissue in a homogeneous manner all culminate in variance within the dataset (Rogers et al., 2018). In particular, even rigorous homogenization of tissue to release PNN components into solution proved difficult, as western blots were highly variable across cases and within a case for the different PNN component levels. As such, other strategies will need to be considered for quantifying amount of expression between cases. We quantified regions (DN, GPi, and GPe) as a whole, so while we did not observe decreases in neuronal number within the DN, the results could be different if the area was subdivided into functional regions based on input from ASD affected areas in the cerebellar hemisphere. Since we did not have access to sections throughout the entire brain region of interest, true stereological assessments were not possible and will be useful for future studies when consistently fixed regions throughout an entire structure are available. While we observed significant density decreases in PV projection neurons with PNNs in the GP, it will be important to consider how this difference intersects with volume differences of the GP in ASD. Again, studies where the entire span of the GP can be dissected and therefore the total area of each region can be quantified and sampled unbiasedly will provide more definitive assessments of neuron density as compared to volume changes.

## Discussion

Despite reports of significant reductions in the number of PCs in the lateral hemisphere of the cerebellum, the targets of PC output within the DN appear to be sustained by the remaining PCs and inferior olive input. Individual neurons within the DN are each contacted by hundreds of PCs (Chan-Palay, 1973; Baumel et al., 2009) so it is reasonable that loss of a percentage of PCs would not impact numbers of DN neurons. The proportion of DN neurons surrounded by PNNs compared to those without appear to be unaltered as well. Future work may reveal differences in expression of PNN components that may be dependent on activity, but the variability inherent to current human postmortem methods did not allow for comparison of expression levels with IHC or western blots (see section “Limitations”).

Within the GP, PNNs mainly surround PV positive projection neurons. This is in contrast to the DN neurons, in which PNNs mainly surround excitatory projection neurons that have input from PV positive PCs. Therefore, differences in ASD could be due to the different functional roles of these neurons subtypes. Both the GPi (with a higher percentage of PNNs) and the GPe had lower density counts in ASD tissue. It is typical for postmortem ASD studies to include seizure status as a covariate, as seizures can lead to many alterations and may be treated with medications that can affect the components being analyzed. One study measured PNN degradation and found that seizures lead to shifts in expression of PNN components, including HPLN1 (Mcrae et al., 2012), which may be due to release of matrix metalloproteinases following status epilepticus (Dzwonek et al., 2004; Dubey et al., 2017). However, a human postmortem study of PNN degradation after epilepsy did not show dysregulation of PNNs (Rogers et al., 2018), which may be dependent on the PNN markers examined and timing following seizures (reviewed in Chaunsali et al., 2021). Consequently, it is not clear whether PNNs degrade similarly in humans or whether the degradation is transient and potentially stabilizes over time.

Since the basal ganglia in general has been reported to have volume differences (for review: Subramanian et al., 2017) and receptor differences (Brandenburg et al., 2020) in ASD, the alterations in PNN density could be due to a general phenomenon of altered activity-dependent mechanisms. Emerging research implicates PV network dynamics in both critical period plasticity and adult learning, where experience related plasticity modulates learning and is dependent on a high or low PV network configuration (Hensch, 2005; Donato et al., 2013). Given that PNNs regulate input onto the soma of PV positive neurons, their digestion with chondroitinase ABC can alter this input and have effects on gamma oscillations, a key component of the role of PV neurons in network dynamics (Carceller et al., 2020). The development of complex skills, such as language and social communication, may be dependent on the function of PV neurons and PNNs in the temporal alignment of critical periods of plasticity across brain regions (Carulli and Verhaagen, 2021). Accumulating evidence implicates vulnerability of PV neurons in neuropsychiatric conditions (Ruden et al., 2021), making them intriguing targets for research and treatment strategies in ASD. The current study suggests alterations in PV neurons within the GP, which warrants further investigations into the function of these specialized neurons, as they are projection neurons that likely have different physiological functions compared to the typically studied PNN positive PV interneurons in cortical regions. As restricted and repetitive behaviors are core symptoms of an ASD diagnosis and the BG is a key regulator of repetitive behaviors, targeting these cell types could be valuable in mitigating undesirable symptoms.

## Data Availability Statement

The original contributions presented in the study are included in the article/supplementary material, further inquiries can be directed to the corresponding author.

## Ethics Statement

Ethical review and approval was not required for the study on human participants in accordance with the local legislation and institutional requirements. Written informed consent for participation was not required for this study in accordance with the national legislation and the institutional requirements.

## Author Contributions

CB: conceptualization and investigation. CB and GB: methodology, writing – original draft, writing – review and editing, and funding acquisition. GB: supervision. Both authors contributed to the article and approved the submitted version.

## Conflict of Interest

The authors declare that the research was conducted in the absence of any commercial or financial relationships that could be construed as a potential conflict of interest.

## Publisher’s Note

All claims expressed in this article are solely those of the authors and do not necessarily represent those of their affiliated organizations, or those of the publisher, the editors and the reviewers. Any product that may be evaluated in this article, or claim that may be made by its manufacturer, is not guaranteed or endorsed by the publisher.
